# A comparative analysis of costs of single and dual rapid HIV and syphilis diagnostics: results from a randomised controlled trial in Colombia

**DOI:** 10.1136/sextrans-2016-052961

**Published:** 2017-05-11

**Authors:** Carol Dayo Obure, Hernando Gaitan-Duarte, Ricardo Losada Saenz, Lina Gonzalez, Edith Angel-Muller, Maura Laverty, Freddy Perez

**Affiliations:** 1 Department of Global Health and Development, Faculty of Public Health and Policy, London School of Hygiene and Tropical Medicine, London, UK; 2 Obstetrics and Gynecology Department, Universidad Nacional de Colombia, Bogotá, Colombia; 3 Department of Reproductive Health and Research, World Health Organization, Geneva, Switzerland; 4 Communicable Diseases and Health Analysis Department, HIV, Hepatitis, Tuberculosis and Sexually Transmitted Infections Unit, Pan American Health Organization, Washington DC, USA

**Keywords:** ANTENATAL HIV, LATIN AMERICA, SYPHILIS

## Abstract

**Background:**

HIV and congenital syphilis are major public health burdens contributing to substantial perinatal morbidity and mortality globally. Although studies have reported on the costs and cost-effectiveness of rapid diagnostic tests (RDTs) for syphilis screening within antenatal care in a number of resource-constrained settings, empirical evidence on country-specific cost and estimates of single RDTs compared with dual RDTs for HIV and syphilis are limited.

**Methods:**

A cluster randomised controlled study design was used to compare the incremental costs of two testing algorithms: (1) single RDTs for HIV and syphilis and (2) dual RDTs for HIV and syphilis, in 12 health facilities in Bogota and Cali, Colombia. The costs of single HIV and syphilis RDTs and dual HIV and syphilis RDTs were collected from each of the health facilities. The economic costs per woman tested for HIV and syphilis and costs per woman treated for syphilis defined as the total costs required to test and treat one woman for syphilis were estimated.

**Results:**

A total of 2214 women were tested in the study facilities. Cost per pregnant woman tested and cost per woman treated for syphilis were US$10.26 and US$607.99, respectively in the single RDT arm. For the dual RDTs, the cost per pregnant woman tested for HIV and syphilis and cost per woman treated for syphilis were US$15.89 and US$1859.26, respectively. Overall costs per woman tested for HIV and syphilis and cost per woman treated for syphilis were lower in Cali compared with Bogota across both intervention arms. Staff costs accounted for the largest proportion of costs while treatment costs comprised <1% of the preventive programme.

**Conclusions:**

Findings show lower average costs for single RDTs compared with dual RDTs with costs sensitive to personnel costs and the scale of output at the health facilities.

**Trial registration number:**

NCT02454816; results.

## Introduction

HIV and syphilis infection contribute to substantial perinatal morbidity and mortality worldwide. WHO and the Joint United Nations Programme on HIV/AIDS estimates that each year 150 000 (110 000–190 000) infants are born with HIV^1^ and 350 000 perinatal deaths are caused by untreated maternal syphilis at the global level.^2^ The prevalence of gestational syphilis in Latin America and the Caribbean varies by country from 0.08% to 7.0%.^3^ In 2015, the incidence of congenital syphilis in Colombia was 3.86 per 1000 live births and the HIV mother to child transmission rate reported as 3.8%. However, only 62% of pregnant women were tested for syphilis during this period in Colombia.^4^

A number of regional and global initiatives have been launched for the dual elimination of mother-to-child transmission of HIV and syphilis.^5–7^ As part of these efforts, provision of screening and treatment within antenatal care (ANC) services have been promoted as an effective and cost-efficient way to deliver services and reduce the risk of mother to child transmission of HIV and syphilis while improving pregnancy outcomes.^8^ ^9^

In resource-constrained settings, many syphilis and HIV infections go undiagnosed and women are lost to follow-up as diagnosis can only be confirmed after laboratory diagnosis which in many cases takes more than a day.^10^ As a result, treatment is delayed and some women never receive treatment. Improvements in syphilis and HIV screening have been made possible by the introduction of rapid diagnostic tests (RDTs) that use finger-prick whole blood samples and do not require laboratories that are unavailable in many lower level facilities.^11^ In these settings, RDTs can be used to allow for early diagnosis and treatment of syphilis using penicillin during a single visit. Combining the delivery and implementation of HIV and syphilis services, using dual rapid tests for screening could be useful for allowing reducing testing barriers and increasing uptake of testing for both HIV and syphilis. Additional advantages of dual HIV/syphilis RDTs may include streamlined procurement, minimised storage space and simplified training of healthcare personnel.^12^

Dual rapid HIV and syphilis tests reported sensitivity and specificity estimates comparable to single HIV and syphilis tests. A recent evaluation of the SD Bioline HIV/Syphilis Duo test conducted in Peru found that the sensitivity for the HIV component of the dual HIV/syphilis test was 99.1% (95% CIs 94.8% to 100%) and the specificity of the HIV component was 99.4% (95% CI 97.7% to 99.9%).

In Colombia, prior to 2014, syphilis screening during ANC visits was mainly done using non-treponemal tests, while HIV diagnosis was done with two positive enzyme immunoassay (EIA) tests (or through a rapid test in places where EIA tests were not available) and confirmed by western blot analysis.

Although a number of studies have reported on the costs of RDTs for syphilis screening in a number of resource-constrained settings,^13–15^ empirical evidence on country-specific cost estimates of single and dual RDTs for syphilis and HIV are limited particularly in low prevalence settings. Empirical evidence on the costs of single and dual RDTs is required to improve delivery of HIV and syphilis screening and health outcomes as well as for programme planning and budgeting purposes.

The objective of this study was to compare the costs of single and dual RDTs for HIV and syphilis in Colombia.

## Methods

### Study setting

A cluster randomised controlled trial was conducted in 12 health facilities (4 hospitals and 8 health centres) in Bogota and Cali, Colombia. Clusters were selected through convenience sampling by choosing health facilities based on a history of at least 250 first antenatal visits per month and similar reported number of cases for HIV and syphilis. Six clusters were selected in each city and randomly allocated to the single HIV syphilis RDT arm (arm A) or dual HIV and syphilis RDT arm (arm B) with a 1:1 allocation ratio using SAS software (release 9.3). No other matching of facilities was conducted apart from the number of first antenatal visits per month. Enrolment to the study was conducted between October 2014 and April 2015 and a total of 2214 pregnant women were enrolled.

### Ethical approval

This study is part of an overall assessment of the feasibility, effectiveness and costs of introducing single and dual RDTs for syphilis and HIV in ANC services in Colombia.^16^ Ethical approval for this study was obtained from the National University of Colombia and the WHO Ethics Research Committee. The identifier on the international randomised controlled trials register is NCT02454816.

### Cost analysis

A prospective costing study was undertaken from the health system perspective (patient out of pocket costs were excluded) using a combination of standard step-down and ingredient-based costing approaches.^17^ The ingredients-based costing approach requires the identification and specification of each component of resource used for delivering an individual service to arrive at a total unit cost. The step-down costing method is used to allocate shared and overhead costs or resources that serve different services or activities.^18^

A standardised excel-based cost sheet was used to obtain data on output and cost information associated with the provision of syphilis and HIV testing for women accessing first ANC visits using single and dual RDTs for a 6-month period between October 2014 and March 2015. The main activities of the HIV and syphilis screening provided as part of ANC were defined and the resources used to test and treat women described in all health facilities were included in the study. These consisted of start-up activities (including development of information, education and communication materials (IEC) and training of health workers), diagnostics and treatment.

Economic costs were estimated which included the value of all resources used to produce output including those for which there were no financial transactions such as volunteer human resources and donated goods.

Costs were divided into capital and recurrent costs. Capital costs comprised the one-off costs of developing IEC materials and training of health workers. Recurrent costs comprised personnel, diagnostics and treatment of syphilis and quality assurance. Personnel costs were calculated based on monthly salaries and allowances and proportion of time spent on syphilis and HIV testing activities. These costs were determined through observational time and motion studies implemented in all health facilities. Time units were multiplied by the relevant clinical staff salaries to obtain total personnel costs for the period of the study.

For diagnostic costs, quantities of supplies and the test kits required to conduct syphilis and HIV tests were obtained from the health workers and multiplied by the unit price to obtain unit cost per diagnostic test for syphilis and HIV test in each intervention arm. The rapid tests used in single RDT arm (singles tests for HIV/syphilis) were: SD Bioline syphilis 3.0 and SD Bioline HIV-1/2 3.0. In the dual RDT arm, the dual test SD Bioline HIV/Syphilis Duo was used. Both tests were provided by the same manufacturer, Standard Diagnostics, Korea. The cost of the RDTs were US$1.03 for the single syphilis test; US$1.23 for the single HIV test and US$3.62 for the dual HIV and syphilis test. Total diagnostic costs were then estimated by multiplying the unit cost per test by the number of pregnant women tested.

Treatment costs for syphilis were estimated based on the standard recommended treatment for syphilis in pregnant women using three 2.4 million units (MU) weekly doses of benzathine penicillin for a total of 7.2 MU. Total treatment costs were obtained by multiplying the number of penicillin doses by the number of women who received treatment for syphilis at the health facility. Treatment of HIV was not considered as pregnant women testing positive for HIV were referred to the tertiary hospital for confirmation of the diagnosis and treatment. Costs of treatment for congenital syphilis were not considered as the pregnant women were not followed-up to birth to determine the perinatal outcome.

Quality assurance costs reflected regular testing of known positive and negative samples at the reference laboratory to evaluate the accuracy of the test kits. Cost components included transport of samples to the central laboratory, monitoring and supervision, with external quality control assessment conducted.

All cost data were collected in Colombian Pesos and converted to US$2015 using an exchange rate of US$1.00=2, 353.95 Colombian Pesos.

### Health outputs

Study outputs included number of pregnant women tested for HIV and syphilis in each of the facilities, number of women testing positive for syphilis and HIV and number of women treated for syphilis. Output data were collected over the same period as the costs. The economic unit cost per woman tested and the cost per woman treated were both calculated for each study facility. The average cost per woman tested was calculated by dividing the total cost of testing by the number of women tested. The average cost per woman treated for syphilis defined as the total costs of resources required to test and treat one woman for syphilis was calculated by dividing the total costs of testing and treatment by the number of women treated.

## Results

### Staff time estimates

The time and motion results revealed average time estimates of 25 min (range 21–29 min) in the single test arm and 29 min (range 22–44 min) in the dual rapid test arm. Additional information on the time estimates for ANC activities and syphilis and HIV testing across intervention arms during the intervention period and a summary of the time estimates for each study facility are provided in the online supplementary file 1.

10.1136/sextrans-2016-052961.supp1 supplementary file

### Output measures

A total of 2214 women attending first ANC visit were tested for HIV and syphilis in the study (table 1). Of the 1048 women tested in the single RDT arm, 3 (0.28%) tested positive for HIV and 29 (2.8%) tested positive for syphilis. Of those who tested positive for syphilis, 24/29 women (83%) received timely treatment. In the dual rapid test arm, 1166 women were tested for HIV and syphilis. Of the women tested, 5 (0.42%) tested positive for HIV and 20 (1.7%) tested positive for syphilis; 20/20 (100%) of the women who tested positive for syphilis received timely treatment.

**Table 1:** Total number of women tested for HIV and syphilis and treated for syphilis by intervention arm

	Single RDT	Dual RDT	Total
Number of pregnant women tested	1048	1.166	2214
Number of HIV reactive tests	3 (0.28%)	5 (0.42%)	8 (0.36%)
Number of syphilis reactive tests	29 (2.8%)	20 (1.7%)	49 (2.21%)
Number of women treated for syphilis	24 (83%)*	20 (100%)	44 (89.8%)

*Five patients did not receive timely treatment due to lack of adherence to protocol by the physicians.

RDT, rapid diagnostic test.

#### Total costs

Table 2 presents the total facility economic costs and average unit costs per woman for testing and treatment of syphilis by treatment arm and health facility level. Average total economic costs were US$1847.99 for the single RDT arm for a total population of 1048 pregnant women across the six facilities. For the dual RDT arm, average total economic costs were US$3074.43 for a total population of 1166 pregnant women across the six facilities. In the single RDT arm, total average costs were higher in the health centres compared with the hospital while in the dual RDT arm, total average costs were higher in the hospitals.

**Table 2:** Total incremental costs for screening and treatment by treatment arm and health facility level

	Single RDT arm			Dual RDT arm		
	Hospital (n=1)	Health centre (n=5)	All	Hospital (n=3)	Health centre (n=3)	All
Start-up costs*	US$139.48	US$132.67	**US$133.81**	US$127.31	US$142.63	**US$134.97**
Clinical staff salaries	US$737.16	US$1117.23	**US$1053.89**	US$2396.26	US$1323.15	**US$1859.71**
Diagnostics†	US$725.70	US$590.56	**US$613.09**	US$1090.32	US$973.50	**US$1031.91**
Treatment‡	US$14.10	US$12.04	**US$12.39**	US$5.90	US$12.98	**US$9.44**
Quality assurance	US$38.09	US$34.16	**US$34.81**	US$43.68	US$33.12	**US$38.40**
**Total costs**	**US$1654.59**	**US$1886.67**	**US$1847.99**	**US$3663.48**	**US$2485.39**	**US$3074.43**
**Cost per woman tested**	**US$8.10**	**US$10.70**	**US$10.26**	**US$17.73**	**US$14.04**	**US$15.89**
**Cost per woman treated**	**US$413.65**	**US$646.86**	**US$607.99**	**US$2958.45**	**US$760.07**	**US$1859.26**

*Includes training costs.

†Diagnostics includes the costs of the test kits and supplies used for testing.

‡Based on three doses of benzathine penicillin.

RDT, rapid diagnostic test.

Bold: Average cost.

**Figure 1:**
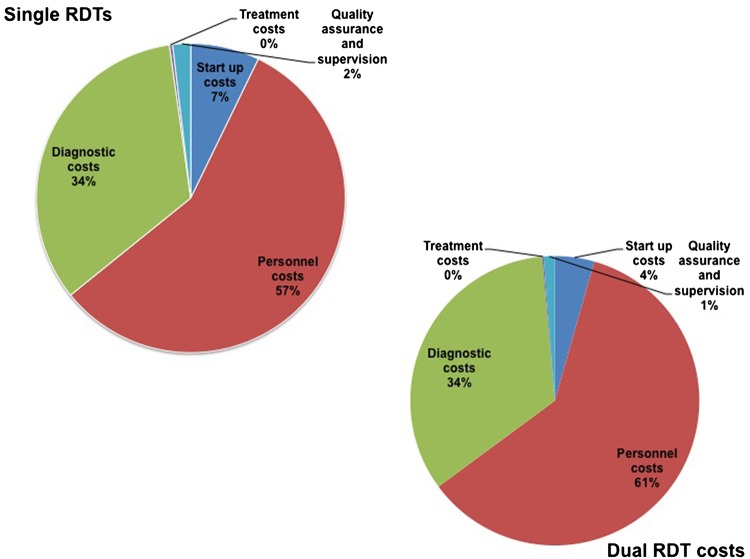
Breakdown of total costs by input type across intervention arms. RDT, rapid diagnostic test.

When total economic costs were analysed by input type (figure 1), clinical staff costs accounted for the largest proportion of total costs across both arms (57% in the single RDT arm and 61% in the dual RDT arm). Diagnostics were the next largest cost category accounting for 34% of total costs in both intervention arms. Start-up costs accounted for approximately 7%–4% of total costs in the single RDT and dual RDT arms, respectively. The quality assurance costs reported reflect the quality assurance implemented during the study, which accounted for 2% of total costs in the single RDT arm and 1% of total costs in the dual arm RDT arm. Treatment costs using 2.4 MU weekly doses for a total of 7.2 MU comprised a very small proportion of total costs accounting for <1% of total costs in both arms.

#### Average unit costs

The cost per woman tested using single RDTs at the health facility level were US$10.26 per pregnant woman tested varying across health facility type from US$8.10 in the hospital to US$10.70 in the health centres. Average cost per woman treated for syphilis was US$607.99 varying between US$413.65 in the hospital and US$646.86 in the health centres. For the dual RDT arm, average cost per woman tested was US$15.89 varying across facility types between US$14.04 in the health centres and US$17.73 in the hospitals and US$1859.26 per woman treated for syphilis varying between US$760.07 in the health centres and US$2958.45 in the hospitals.

Table 3 presents a breakdown of the cost per woman tested by input type. Unit start-up costs ranged from US$0.62 to US$0.84. Unit costs for clinical staff salaries accounted for the largest proportion of unit cost across both intervention arms. Unit costs for the test kits and other supplies were estimated at US$3.54 in the single RDT arm and US$5.31 in the dual RDT arm. Unit costs for quality assurance were US$0.21 and US$0.19 in Bogota and Cali, respectively.

**Table 3:** Breakdown of cost per woman tested in US$2015

	Single RDT arm		Dual RDT arm	
	Hospital (n=1)	Health centre (n=5)	Hospital (n=3)	Health centre (n=3)
Start-up costs*	$0.68	$0.75	$0.62	$0.84
Clinical staff salaries	$3.69	$6.02	$11.58	$7.77
Diagnostics†	$3.54	$3.54	$5.31	$5.31
Quality assurance	$0.19	$0.19	$0.21	$0.14
Cost per woman tested	$8.10	$10.70	$17.72	$14.06

*Includes training costs.

†Diagnostics includes the costs of the test kits and other supplies used for testing.

RDT, rapid diagnostic test.

## Discussion

This study uses observation data from clinic-based evaluations to determine the costs of single and dual RDTs for HIV and syphilis among pregnant women attending ANC in Bogota and Cali, Colombia. While a number of studies have reported the costs of HIV and syphilis testing in high prevalence settings, this study focuses on a low prevalence setting and is the first to present a comparative cost analysis of the single and dual HIV and syphilis testing in Colombia.

The study found the average unit cost at the health facility level for HIV and syphilis testing within ANC to be US$10.26 per woman tested and US$607.99 per woman treated for syphilis using the single RDT. This was lower than the estimated unit cost for the dual RDT arm at US$15.89 per pregnant woman tested and US$1859.26 per woman treated for syphilis. Our unit costs varied widely across health facilities for both testing arms.

The estimated costs also varied between the two cities. Average cost per woman tested and average costs per woman treated for syphilis were higher in Bogota for both arms compared with Cali. The considerable variation in costs per woman tested across cities suggests a potential to improve cost efficiency across health facilities through better allocation of existing personnel where staff costs are high and workload is low. In many of the study facilities, particularly the study sites in Bogota where the syphilis and HIV incidences were lower than Cali, the staff workload per day was low and did not warrant a dedicated staff allocated to providing HIV and syphilis testing services only.

The lower unit costs in Cali also support the hypothesis that costs per woman treated for syphilis are highly dependent on the epidemiology of the disease. Unit costs rise dramatically where syphilis prevalence is low as there are few women to be treated for syphilis. However, results from studies suggests that expanding syphilis screening and treatment into ANC programmes is cost saving or highly cost-effective in settings with high maternal syphilis prevalence, low current service coverage and high healthcare cost as well as in low maternal syphilis prevalence settings.^19–21^

This study found higher costs per woman tested using dual RDTs compared with single RDTs across both cities in Colombia. While counterintuitive, this result can be explained by the higher diagnostic test kit cost for the dual test and higher personnel costs in the sites randomised to the dual RDT arm. One logical explanation for the high personnel costs in the dual RDT sites is the relative size of the health facilities given that three of the study sites were secondary facilities with higher staff costs. In addition, the higher personnel costs are supported by underlying data obtained from the time and motion studies which reveal higher time estimates for testing using the dual RDTs (average 29 min) compared with the single RDTs (average 25 min).

The unit costs derived from this study are higher than those reported in a recent study conducted in Peru, another low syphilis and HIV prevalence setting.^20^ The Peruvian study found cost estimates ranging from US$2.70 to US$3.19 and US$295 to US$369 per woman tested with rapid single test (RST) compared with US$3.60 to US$5.55 and US$740 to US$1454 per woman tested with rapid plasma reagin (RPR). The lower costs reported for the Peruvian study is largely a reflection of the presence of economies of scale in implementation where fixed costs such as personnel are spread over a large volume of output. Overall, the high average costs per woman tested and treated in this present study can be attributed to the low numbers of women testing positive for syphilis in this setting.

Like any other costing study, this study has a number of limitations that should be mentioned. First, we acknowledge the possible drawback of matching using a convenience sample, which may have caused biased research results and thus limiting the wider applicability of the study results. Second, although HIV testing was provided in the context of this study, the study did not attempt to estimate the costs of HIV treatment as patients who tested positive were referred to a central location for antiretroviral treatment (ART) treatment. Third, no effectiveness measures were obtained from the trial limiting the ability to conduct a cost-effectiveness analysis. As there was no retesting of samples with a reference standard, the true prevalence of syphilis in the study facilities is unknown. Further research is therefore required to determine the cost-effectiveness of the two testing algorithms in Colombia.

## Conclusion

As rapid test for HIV and syphilis are rolled out, this study provides valuable information for policymakers and public health practitioners seeking to implement rapid diagnostics for syphilis and HIV screening programmes in ANC settings in Colombia and other low prevalence settings. The results of the study show lower average costs for single RDTs compared with dual RDTs. However, it is worth noting that if the price of the dual RDTs were lowered due to increased demand, the unit costs per woman tested would likely reduce. In conclusion, although this study finds higher unit costs for dual RDTs compared with single RDTs, other considerations beyond costs should be taken into account when making decisions on the roll out of rapid diagnostics for HIV and syphilis.

**Key messages**

- Average unit costs for single rapid diagnostic tests (RDTs) were lower compared with dual RDTs in the study sample.
- Human resource costs are the major cost driver across the two testing strategies.
- Other considerations beyond costs such as increased testing uptake for both HIV and syphilis should be taken into account by decision makers.
