# Supplementary material for: A comparative analysis of costs of single and dual rapid HIV and syphilis diagnostics: results from a randomised controlled trial in Colombia
Source: Sex Transm Infect. 2017 May 11;93(7):482–6. doi: 10.1136/sextrans-2016-052961 (PMC5739853; doi:10.1136/sextrans-2016-052961)
Supplement: supplementary file [file sextrans-2016-052961supp001.pdf]

Annex 1: Time and Motion Study

Table 1: Time and motion data (Average time estimates from observations at health facilities)

Health facility	Intervention Arm	Location	Health facility type	Patient registration (mins)	Clinical examination (mins)	Counseling for HIV/syphilis (mins)	Collecting blood for screening (mins)	Sample testing (mins)
CAMI Bosa Povenir	Single RDT	Bogota	Health Centre	28.09	13.73	12.33	1.58	15.00
CAMI Altamira	Single RDT	Bogota	Health Centre	23.73	14.53	11.00	1.47	15.00
UPA Primera de Mayo	Single RDT	Bogota	Health Centre	21.50	12.00	11.00	1.67	15.20
El Vallado	Single RDT	Cali	Health Centre	24.43	9.50	9.36	1.00	15.00
Marroquin	Single RDT	Cali	Health Centre	15.00	9.93	5.00	1.00	15.00
Carlos Holmes Trujillo	Single RDT	Cali	Hospital	33.43	8.57	5.36	2.00	15.00
Decepaz	Dual RDT	Cali	Health Centre	12.50	5.00	4.86	2.00	15.00
El Diamante	Dual RDT	Cali	Health Centre	20.00	9.45	7.86	1.14	15.00
Potrero Grande	Dual RDT	Cali	Health Centre	22.14	9.43	5.57	2.00	15.00
Pablo VI	Dual RDT	Bogota	Hospital	18.75	13.25	16.73	1.60	15.00
CAMI Suba	Dual RDT	Bogota	Hospital	21.67	14.47	24.73	1.27	18.33
Vista Hermosa	Dual RDT	Bogota	Hospital	25.73	13.09	11.86	1.14	15.00

Table 2: Average time estimates for HIV and Syphilis testing (in minutes) by Intervention arm

**Single Rapid Test Arm (Arm A)**		**Dual Rapid Test Arm (Arm B)**	
CAMI Altamira Health Center	27.47	CAMI Suba Hospital	44.33
CAMI Bosa Porvenir Health Center	28.92	Decepaz Health Center	21.87
Carlos Holmes Trujillo Hospital	22.33	El Diamante Health Center	23.93
El Vallado Health Center	25.20	Hospital Pablo VI Hospital	33.33
Marroquín Health Center	21.00	Hospital Vista Hermosa Hospital	28.20
UPA Primera de Mayo Health Center	27.87	Potrero Grande Health Center	22.53
**Average time**	**25 minutes**		**29 minutes**
